# First report of begomoviruses infecting *Cucumis sativus* L. in North America and identification of a proposed new begomovirus species

**DOI:** 10.7717/peerj.9245

**Published:** 2020-07-10

**Authors:** Sarahi Sanchez-Chavez, Carlos Fernando Regla-Marquez, Zugey Elizabeth Cardenas-Conejo, Daniel Alejandro Garcia-Rodriguez, Sara Centeno-Leija, Hugo Serrano-Posada, Andromeda Liñan-Rico, Brenda Lizet Partida-Palacios, Yair Cardenas-Conejo

**Affiliations:** 1 Laboratorio de Agrobiotecnologia, Universidad de Colima, Colima, Mexico; 2 Laboratorio de Agrobiotecnologia, Consejo Nacional de Ciencia y Tecnologia-Universidad de Colima, Colima, Mexico; 3 Centro Universitario de Investigaciones Biomedicas, Consejo Nacional de Ciencia y Tecnologia-Universidad de Colima, Colima, Mexico

**Keywords:** Geminivirus, Begomovirus, ssDNA virus, High-Throughput Sequencing, Cucumber

## Abstract

**Background:**

Members of the *Begomovirus* genus are phytopathogens that infect dicotyledonous plants, producing economic losses in tropical and subtropical regions. To date, only seven species of begomoviruses (BGVs) infecting cucumber have been described. Most cucumber infections were reported in South Asia. In the Americas, begomoviral infections affecting cucumber are scarce; just one report of begomovirus has been described in South America. The presence of whitefly and typical symptoms of viral infections observed in a cucumber field in Colima, Mexico, suggested that plants in this field were affected by BGVs.

**Methods:**

To identify the BGVs infecting cucumber, we performed a high-throughput sequencing and compared the assembled contigs against the GenBank nucleic acid sequence database. To confirm the presence of viruses in cucumber samples, we performed a PCR detection using specific oligonucleotides. We cloned and sequenced by Sanger method the complete genome of a potential new begomovirus. Begomovirus species demarcation was performed according to the International Committee on Taxonomy of Viruses. The evolutionary relationship of the new virus was inferred using phylogenetic and recombination analyses.

**Results:**

We identified five species of begomovirus infecting plants in a field. None of these have been previously reported infecting cucumber. One of the five species of viruses here reported is a new begomovirus species. Cucumber chlorotic leaf virus, the new species, is a bipartite begomovirus that has distinctive features of viruses belonging to the squash leaf curl virus clade.

**Conclusions:**

The findings here described represent the first report of begomoviral infection affecting cucumber plants in North America. Previous to this report, only seven begomovirus species have been reported in the world, here we found five species infecting cucumber plants in a small sample suggesting that cucumber is vulnerable to BGVs. One of these viruses is a new species of begomovirus which is the first begomovirus originally isolated from the cucumber. The findings of this report could help to develop strategies to fight the begomoviral infections that affect cucumber crops.

## Introduction

The family *Geminiviridae* is a monophyletic group characterized by a twinned capsid structure that all members possess. Members of this family have small genomes (2.5–5.2 kb) composed of one or two circular single-strand DNA which are replicated by a rolling-circle mechanism (Hanley-Bowdoin et al., 1999; Jeske, 2009). Geminiviruses are phytopathogens that cause economic losses by diseases in monocotyledonous and dicotyledonous crops (Rojas et al., 2005). This family contains about 440 species that are classified into nine genera (*Becurtovirus*, *Begomovirus*, *Capulavirus*, *Curtovirus*, *Eragrovirus*, *Grablovirus*, *Mastrevirus*, *Topocuvirus* and *Turncurtovirus*) based on genome organizations, host ranges and insect vector (Zerbini et al., 2017).

The genus *Begomovirus* is the most diverse genus of geminiviruses, more than 409 species of begomoviruses (BGVs) have been described (Zerbini et al., 2017). A wide range of dicotyledonous crops are host of BGVs which are transmitted by the whitefly *Bemisia tabaci* (Hemiptera; Aleyrodidae) (Jeske, 2009). The genome of BGVs is constituted by one (monopartite genome) or two (bipartite genome) genomic components. Components for the bipartite viruses are known as DNA-A and DNA-B. DNA-A is homologous to the genome of monopartite virus; they conserve the genomic structure and genes. The complementary-sense of this genomic component encodes the replication-associated protein (Rep), the replication enhancer protein (REn), the transcription-activator protein (TrAP) and the protein AC4 (AC4) which is involved in the suppression of gene silencing. The virion-sense encodes the coat protein (CP) in all BGVs and the protein AV2 (AV2) which is distinctive of BGVs from the Old World (Lazarowitz & Shepherd, 1992; Jeske, 2009). On the other hand, DNA-B is specialized in the local (nucleus to cytoplasm) and systemic (cell to cell) movement of begomovirus, performed by nuclear shuttle protein (NSP) and movement protein (MP), respectively (Lazarowitz & Shepherd, 1992; Pascal et al., 1994). DNA-A and DNA-B share little sequence identity with the exception of an ~200 bp sequence in the intergenic region (IR) known as the common region (CR) (Hanley-Bowdoin et al., 1999). The CR encompasses the origin of replication, which includes conserved repeated sequences (iterons), required for specific recognition and binding by Rep, and the hairpin structure that harbors the invariant nonanucleotide 5′-TAATATTAC- 3′ whose T^7^–A^8^ site is cleavaged by Rep to begin the replication process (Argüello-Astorga et al., 1994; Hanley-Bowdoin et al., 1999). BGVs may be subdivided into four phylogenetic groups: Old World, New World, legumoviruses and sweepoviruses (Ilyas et al., 2009; Briddon et al., 2010; Trenado et al., 2011). Old World BGVs (originating from Africa, Europa and Asia) can be either mono- or bipartite, while most BGVs from the New World (originating from the Americas) are bipartite and lack the *AV2/V2* gene. New World BGVs are grouped in several secondary linages or “clades,” such as the Squash leaf curl virus (SLCuV) clade, the Abutilon mosaic virus (AbMV) clade and the Brazil clade (Argüello-Astorga et al., 1994; Rojas et al., 2005). Members of the SLCuV clade display distinctive features in their replication origin, AC4 protein and N-terminal domain of their Rep protein (Argüello-Astorga et al., 1994; Gregorio-Jorge et al., 2010; Torres-Herrera et al., 2019). Legumoviruses and sweepoviruses are grouped by the host that they specifically infect. Legumoviruses, present only in the Old World, are restricted to leguminous plants whereas sweepoviruses infect sweet potato and plants of the family *Convolvulaceae* (Briddon et al., 2010).

Cucumber (*Cucumis sativus* L.) is an annual plant species belonging to the family *Cucurbitaceae*. The worldwide annual production of cucumber is about 84 million tons. The main producer countries are China, Iran and Russia (Food and Agriculture Organization of the United Nations, 2017). Apparently, cucumber infections by BGVs are rare in the Americas, since just one report of BGVs affecting cucumber in this region have been described, specifically in Venezuela (Romay et al., 2014). At the time of writing this article, only seven species of BGVs infecting cucumber had been reported, of which six species were identified in South Asia. Interestingly, none of the seven BGVs species that affect cucumber were originally isolated from this crop; they were originally isolated from other crops such as, tomato, mungbean, squash and melon (Ramirez et al., 2004; Mizutani et al., 2011; Shahid et al., 2018; Shafiq et al., 2019).

Colima is a state of Mexico, where the predominant weather in the region is sub-humid which favors cucumber cultivation (Instituto Nacional de Estadística y Geografía, 2016). In a cucumber field in Colima, Mexico, we observed typical symptoms of viral infections and whiteflies feeding on plants, which suggested that a begomoviral infection was affecting the cucumber field. Using an approach that combines rolling circle amplification (RCA) and High-Throughput Sequencing (HTS), which has been used successfully to detected geminiviruses (Idris et al., 2014; Kathurima et al., 2016; Zubair et al., 2017; Rodríguez-Negrete et al., 2019), we found that the cucumber field was affected by five species of BGVs, one of which is a novel species of BGVs.

## Materials and Methods

### Samples collection and DNA extraction

**Figure 1:**
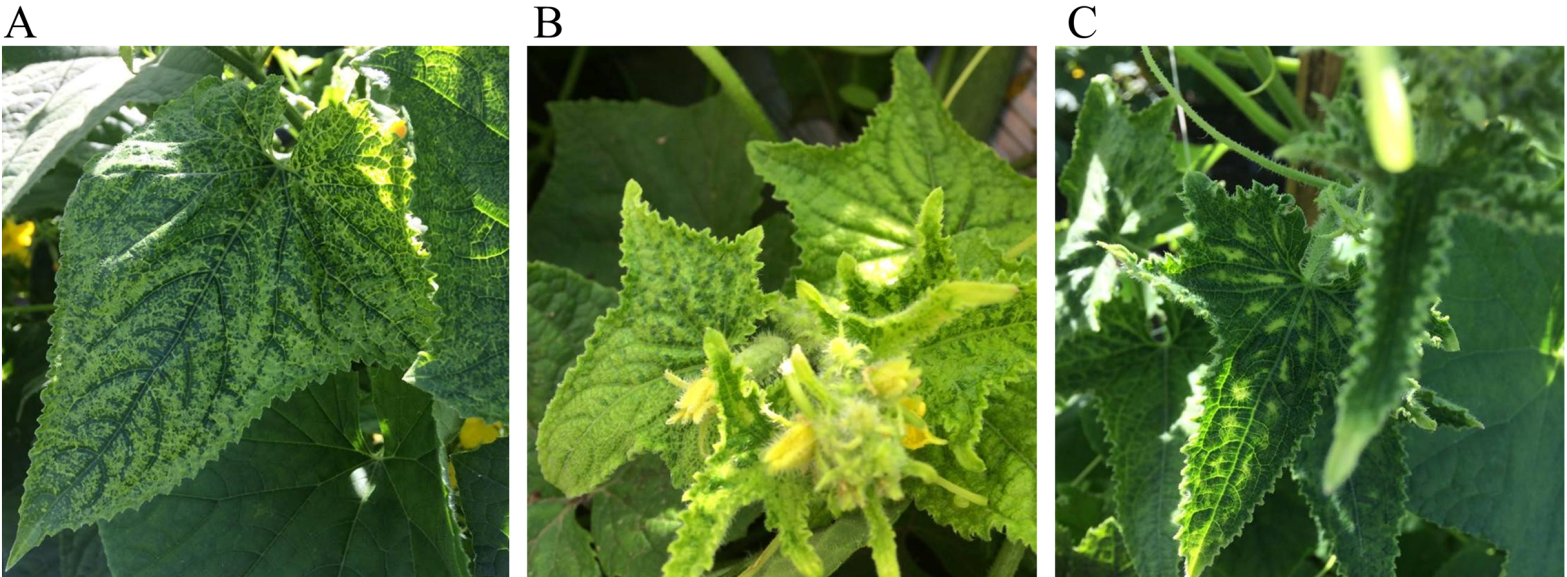
Representative symptoms observed in the cucumber field in Colima, Mexico. The common symptoms that appeared on leaves were chlorosis (A) chlorosis and crinkle (B) and chlorosis associated with leaf curl (C).

Leaves from six cucumber plants with typical begomovirus disease symptoms (Fig. 1), were collected from a field in Colima, Mexico (19°23′07.8″N 103°48′06.8″W) during August 2018. Alejandro Rodriguez, the cucumber plantation owner, gave us verbal permission to access their land and collect samples. The DNA extraction for the collected samples was carried out using the Dellaporta DNA extraction method (Dellaporta, Wood & Hicks, 1983).

### Ilumina sequencing and de novo assembly

Total DNA extracts of each plant sample were used as a template for RCA using phi29 (Thermo Fisher Scientific, Waltham, MA, USA). RCA products (3 µg) from each plant were pooled and 1 µg of the pooled RCA was used as a template for Ilumina sequencing at the Laboratory of genomic services (LABSERGEN, Irapuato, Guanajuato, Mexico). A TrueSeq DNA Nano kit (Illumina Inc., San Diego, CA, USA) was used for the preparation of the library for sequencing. The library was paired-end (PE) sequenced with 150 cycles in one line of the Illumina NextSeq 500 platform (~1 million reads per library), using insert sizes of 480 bp.

Raw data were converted to fastq files, de-multiplexing and adapter removal using bcl2fastq2 Conversion software v2.19.1 (Illumina Inc., San Diego, CA, USA). The quality filter was carried out with Trimmomatic v0.38 (Bolger, Lohse & Usadel, 2014), reads were discarded if the average Phred33 sequence quality (over a 5 bp window) was below 20, and the length of the read was shorter than 50 bp. For quality control checks FastQC v0.11.8-0 and MultiQC v1.6 were used. The assembly process was carried out with SPAdes v3.12.0 using default parameters (Bankevich et al., 2012). To extend SPAdes contigs, CAP3 was used as a super assembler (Huang & Madan, 1999). Contigs <500 bp were not included in the analysis. Mean contig size and N50 statistics were calculated using assemblathon v2 (Bradnam et al., 2013).

### Begomovirus identification

A local BLAST analysis was performed to compare contigs against the GenBank nucleic acid sequence database (nt database), updated on 12 March 2019. The BLASTn algorithm included in the bioinformatic package BLAST+ v2.6.0 (Zhang et al., 2000). An *e*-value cutoff of 1e−20 was used. Positive contigs to BGVs were curated manually to obtain organized sequences in a positive sense and set the nucleotide number one at the nicking site place. Curated contigs were compared again with the nt database to confirm the begomovirus identification.

### Begomovirus PCR detection

To confirm the presence of BGVs identified by HTS, a PCR analysis was carried out using specific primers for DNA-A amplification (Table S1). PCR reaction was carried out in C1000 Thermal cycler (BioRad, Hercules, CA, USA) using standard conditions (1 cycle of 95 °C for 10 min, followed by 32 cycles of 95 °C for 15 s, 56 °C for 30 s and 70 °C for 1 min). Enriched samples (50 ng) were used as template. The reaction mixture consisted of 0.5 µM for each primer (Table S1), 1 U of Dream Taq (M3001, Thermo Scientific^™^, Waltham, MA, USA), 2 μL of 10× Dream Taq buffer, 0.2 mM of each dNTPs in 20 µL of final reaction volume.

### Isolation of complete genome

To obtain the complete sequence of DNA-A of the probably new virus, we selected an infected plant and amplified by PCR two genomic regions denominated upper region (includes the common region and partial AC1 and AV1 genes; coordinates 1819–698) and lower region (includes, partial AC1 and AV1 genes and complete AC2 and AC3 genes; coordinates 614–1958). The upper region was amplified using the primer combination F-Rep_PNA/R-CP_PNA (1,507 bp) and the lower region using the primers R-Rep_PNA/F-CP_PNA (1,345 bp) (Fig. S1A; Table S1).

To obtain the complete sequence of DNA-B, we amplified by PCR the upper region (includes the common region and partial BV1 and BC1 genes; coordinates 1967–821) and the lower region (includes partial BV1 and BC1 genes; coordinates 764–2049). The upper region was amplified using the primer combination F-BC1_NMB/R-BV1_NMB (1,448 bp) and the lower region using the primers R-BC1_NMB/F-BV1_NMB (1,286 bp) (Fig. S1B; Table S1). Subsequently, we cloned the amplicons into pGEM-T easy vector for later sequencing by the Sanger method using the universal M13 primers.

### Genome sequence validation via sanger sequencing

The genome sequence of the new BGV was corroborated by Sanger sequencing. RCA products were digested with single cutting restriction enzymes. DNA-A was linearized with SacI enzyme and cloned into pGEM-T easy (Promega, Madison, WI, USA). DNA-B was linearized with XbaI and cloned into pBlueScript II SK (+) (Addgene, Watertown, MA, USA). Clones were Sanger sequenced using the universal oligonucleotides M13 forward and M13 reverse, as well as F-CP_PNA and R-Rep_PNA for DNA-A and F-BC1_NMB, R-BC1_NMB and F-BV1_NMB for DNA-B. The positions of the primers ensure the full coverage of each genomic component. All clones were sequenced at LANBAMA-IPICYT (San Luis Potosi, Mexico) using a 3130 Genetic Analyzer (Applied Biosystems, Foster City, CA, USA).

### Species demarcation analysis

To perform the demarcation of the probable new species of begomovirus, we followed the steps described by the International Committee on Taxonomy of Viruses (ICTV) (Brown et al., 2015). First, we searched for related BGVs species with the probably new virus using BLASTn tool. The search was directed against the nt database (updated on 12 March 2019) using the search term “txid10814”. Second, we downloaded the complete DNA-A of related sequences in fasta format to create a dataset that includes the complete DNA-A of the probably new virus. All the sequences were curated to set the first nucleotide after the nicking site within the conserved nonanucleotide (taatatt/ac). Third, we aligned the complete DNA-A from the dataset using the MUSCLE algorithm included in the package SDT v1.2 (freely available at http://www.cbio.uct.ac.za/SDT) to calculated identities between every pair of sequences.

### Recombination analysis

Recombination analysis was carried out using the Recombination Detection program version 4 (RDP4), which is a software that applies many recombination detections and analysis methods (Martin et al., 2015). To search for potential parental viruses in the nt database, we used SWeBLAST with a window size of 250 nt and a step size of 100 nt (Fourment, Gibbs & Gibbs, 2008). We considered potential parents to all those viruses that shared an identity percentage greater than 90% within a 250 nts region. Potential parent viruses were aligned using the ClustalW algorithm included in the package MEGA v10.0.4 (Kumar et al., 2018). This alignment was used in the RDP4 program. For all the cases, we used default parameters.

### Phylogenetic analysis

Phylogenetic reconstruction was based on the alignment of 41 DNA sequences from genomic DNA-A of selected New World begomovirus and the Old World virus *Tomato yellow leaf curl virus* as outgroup. Regarding DNA-B, phylogenetic analysis was based on the alignment of 34 DNA-B sequences, and the DNA-B from *African cassava mosaic virus* as outgroup. The two evolutionary histories were inferred by using the maximum likelihood method based on the General Time Reversible model (Nei & Kumar, 2000). Gamma distribution was used to model evolutionary rate with invariable rate variation (G + I). The substitution models were predicted by the Best-Fit substitution model (ML). Phylogeny tests were conducted by the bootstrap method (1,000 replicates). All positions containing gaps and missing data were eliminated. The alignment was performed with the ClustalW algorithm with default parameters. Evolutionary analyses were carried out using algorithms included in MEGA v10.0.4.

### Supporting data

Supporting data are available in NCBI database. Raw data of Illumina sequencing are available in Sequence Read Archive (SRX5908012). The complete genome sequence of the new virus is available in GenBank database; DNA-A (MN013786) and DNA-B (MN013787).

## Results

### Illumina sequencing and de novo sequence assembly of metagenome

In order to detect BGVs in the cucumber field at Colima state, Mexico, we carried out a HTS. After sequencing, we obtained 2,151,838 raw reads, before applying quality filters. Reads with unidentified bases (Ns), average Phred quality below 20 and smaller than 50 bp in length were pulled out. The number of quality reads was 1,738,568 which displayed an average Phred quality of 32.4 and an average length of 134 bp. To assemble the high-quality reads, we used the software SPAdes v3.12.0 with default parameters. To reduce the number of contigs and lengthen them, we re-assembled the resulting contigs with the super assembler CAP3 and pulled out the contigs <500 bp. 17, 557 assembled contigs were obtained (Data S1), which have a mean contig size of 873 bp and N50 of 832 bp.

### BLAST search in public database

To identify the assembled contigs obtained from the cucumber samples, we performed a local BLASTn search against the nt database using the assembled contigs. The BLAST search showed that the hits were distributed in bacteria, plants, viruses and insects. Most contigs had BLAST hits with the host plant while few contigs had hits with viruses, bacteria and insects (Fig. S2; Table S2). All the contigs with bacterial BLAST hits were to *Aureimonas ureilytica* (Table S2).

Nine contigs had hits with viruses that belong to *Begomovirus* genus (Table S2). These nine contigs were curated (Data S2) and compared against the nt database. The BLASTn search using the curated sequences showed that these sequences correspond to the bipartite genome of *Pepper golden mosaic virus* (PepGMV-[pepper mild tigre]), *Pepper huasteco yellow vein virus* (PhYVV-[MX-CL-16]), *Tomato golden mottle virus* (ToGMoV-[MX-SLP-05]) (Table 1). One contig got a hit with the DNA-B of *Rhynchosia golden mosaic Sinaloa virus* (RhGMSV-[MX-SIN-04]), the nucleotide identity percentage was low (Table 1). DNA-A for RhGMSV-[MX-SIN-04] was not identified from the contigs with a length above 500 bp, however, a filter out contig of 461 bp that got a hit with the DNA-A of RhGMSV-[MX-SIN-04] (DQ406672) showed 93% of identity, therefore we can affirm that RhGMSV-[MX-SIN-04] was present in cucumber crop. The pairwise identity displayed by these four BGVs indicated that they are strains of their cognates viruses. The sequences isolated by HST were submitted to the GenBank database (MN013410: PepGMV-DNA-A; MN013411: PepGMV-DNA-B; MN013408: PhYVV-DNA-A; MN013409: PhYVV-DNA-B; MT083928: ToGMoV-DNA-A; MT083929: ToGMoV-DNA-B; MT083930: RhGMSV-DNA-B).

**Table 1:** Begomoviruses identified in cucumber samples.

Contig	BLASTn hits	Component	Nucleotide sequence identity (%)	GenBank Acc. No.
Contig669	PhYVV-[MX-CL-16]	DNA-A (2,170 bp)^1^	99.5	MG582068.1
Contig11		DNA-B	98.26	LN848912.1
Contig523	PepGMV-[pepper mild tigre]	DNA-A	97.5	EF210556.1
Contig534		DNA-B	96.3	LN848807.1
Contig3154	ToGMoV-[MX-SLP-05]	DNA-A	96.6	EF501976.1
Contig555		DNA-B	96.25	DQ406674.1
Contig564	RhGMSV-[MX-SIN-04]	DNA-B	86	DQ406673.1
Contig2983	SbBMV-[AR-NOA-05]*	DNA-A (1,406 bp)^1^	81	EF016486.1
Contig452	ToMHaV-GT-12	DNA-B (2,404 bp)^1^	78.064	KT099164.1

**Notes:**

1 Partial sequence.

* Component A of the potential new virus, according to the pairwise identity threshold (<91) proposed by Brown et al. (2015).

Additionally, we identified the partial DNA-A (1,406 bp) and DNA-B (2,404 bp) sequences of the potential new virus. The BLASTn search showed that partial DNA-A sequence shared 81% of nucleotide identity with *Soybean blistering mosaic virus* isolate NOA (SbBMV-[AR-NOA-05]), while, the partial sequence of DNA-B shared 78% of nucleotide identity with DNA-B of *Tomato mosaic Havana virus* (ToMHV-GT-12) (Table 1).

### PCR detection of begomoviruses identified by HTS

To confirm the presence of the five BGVs identified by HTS in cucumber samples, we carried out a PCR detection using specific primers for each virus (Table S1). PCR analyses confirmed the presence of the five BGVs in cucumber samples (Fig. S3). These findings represent the first report of PepGMV, ToGMoV, PhYVV and RhGMSV infecting cucumber plants. Also, we identified the probably new virus using specific primers (F-CP_PNA/R-Rep_PNA: 1,345 bp). The PCR revealed that all the tested plants were infected with the probable new virus (Fig. S3E). Interestingly, the six tested plants were co-infected with the probable new virus (Fig. S3) and at least with three viruses; this suggests that cucumber is vulnerable to mixed infections of BGVs.

### Full-length genome sequencing for the potential new virus

Since a partial DNA-A sequence from cucumber metagenome showed low nucleotide identity with SbBMV-[AR-NOA-05], we decided to isolate the complete genome sequence. To complete the sequence of DNA-A, we selected an infected plant and amplified by PCR two genomic regions denominated upper and lower regions. The upper region was amplified using the primer combination F-Rep_PNA/R-CP_PNA (1,507 bp) and the lower region using the primers R-Rep_PNA/F-CP_PNA (1,345 bp) (Fig. S4). Subsequently, we cloned the amplicons into pGEM-T easy for sequencing by Sanger method using the universal M13 primers. Sanger sequencing allowed us to assemble the complete DNA-A sequence of the presumably new virus. A comparison between the partial DNA-A sequence from Illumina sequencing and the complete DNA-A obtained for Sanger sequencing showed that both sequences shared 99% identity. The complete DNA-A sequence is 2,627 bp in length. The entire DNA-A was compared against the nt database, the best hits were obtained with *Bean leaf crumple virus* (BLCrV; NC_043524) a BGV that infects common bean in Colombia (Carvajal-Yepes et al., 2017), the coverage was 96% and the pairwise identity was 82%, indicating that DNA-A probably belongs to a new species of begomovirus.

To complete the sequence of DNA-B, we followed a similar strategy used for the DNA-A. The upper region was amplified using the primer combination F-BC1_NMB/R-BV1_NMB (1,448 bp) and the lower region using the primers R-BC1_NMB/F-BV1_NMB (1,286 bp) (Fig. S4). After Sanger sequencing using M13 primers we assembled the complete DNA-B which is 2,599 bp in length. The best hit was obtained with ToMHV-GT-12 (query coverage: 62%; pairwise identity: 78%) when the complete DNA-B was compared against the nt database. To corroborate the sequence and avoid artifactual results by the PCR amplification, we cloned the complete DNA-A and DNA-B produced by restricting RCA products and sequenced the full-length components via Sanger method. Complete DNA-A (MN013786.1) and DNA-B (MN013787.1) were deposited in the GenBank database.

### Species demarcation analysis

To establish if the isolated BGV belongs to a new species, we followed the steps proposed by the ICTV (Brown et al., 2015). For the demarcation process, we selected the first 250 DNA-A sequences resulting from BLASTn search and aligned them by MUSCLE algorithm, included in SDT v1.2 software. Demarcation steps revealed that the highest pairwise identity reached was 82.5% with BLCrV (Table S3); this result confirms that the new virus belongs to the genus *Begomovirus* and that it represents a new species. We propose the name Cucumber chlorotic leaf virus for the new BGV species (abbreviated, CuChLV) and have submitted a taxonomic proposal to the ICTV proposing the new species.

### Genomic characterization of *cucumber leaf curl virus*

**Figure 2:**
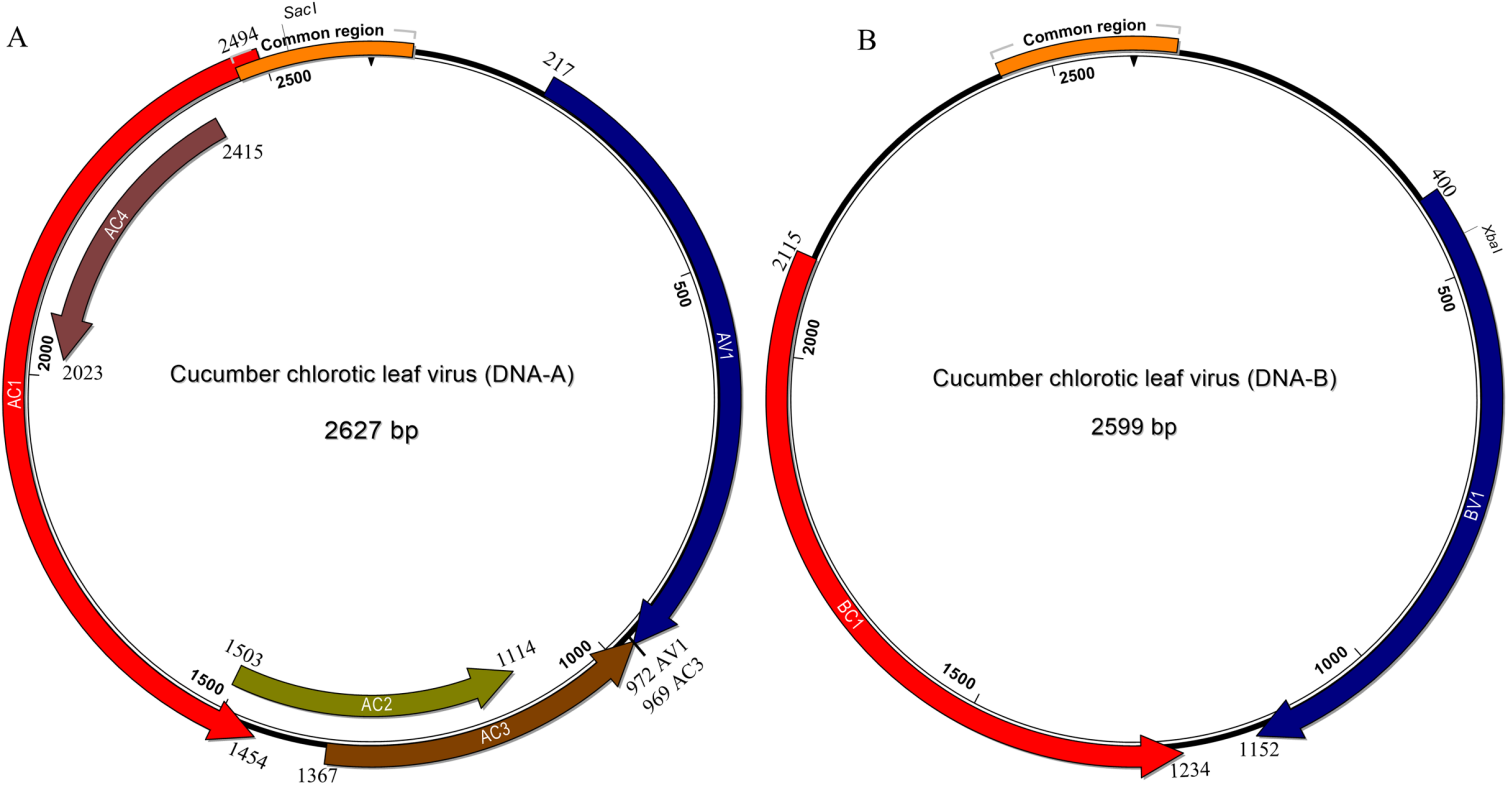
Genomic organization of Cucumber chlorotic leaf virus. Genomic component DNA-A is 2,627 bp in length (A) and DNA-B is 2,599 bp in length (B). Arrows represent the open reading frames of CuChLV. The common region is shown as an orange box. The restriction enzymes SacI and XbaI used to clone the complete DNA-A and DNA-B, respectively are shown.

In common with most begomovirus originating from the New World, the genome of CuChLV is bipartite. The DNA-A contains the typical five genes that encode the proteins CP (gene AV1), Rep (gene AC1), TrAP (gene AC2), REn (gene AC3) and the AC4 (gene AC4). The DNA-A does not have the AV2 gene, typical of BGVs from the Old World (Fig. 2A). On the other hand, DNA-B contains the two genes that encode NSP (gene BV1) and MP (gene BC1) proteins (Fig. 2B). All the proteins have the average size of BGVs proteins, with the exception of AC4.

**Figure 3:**
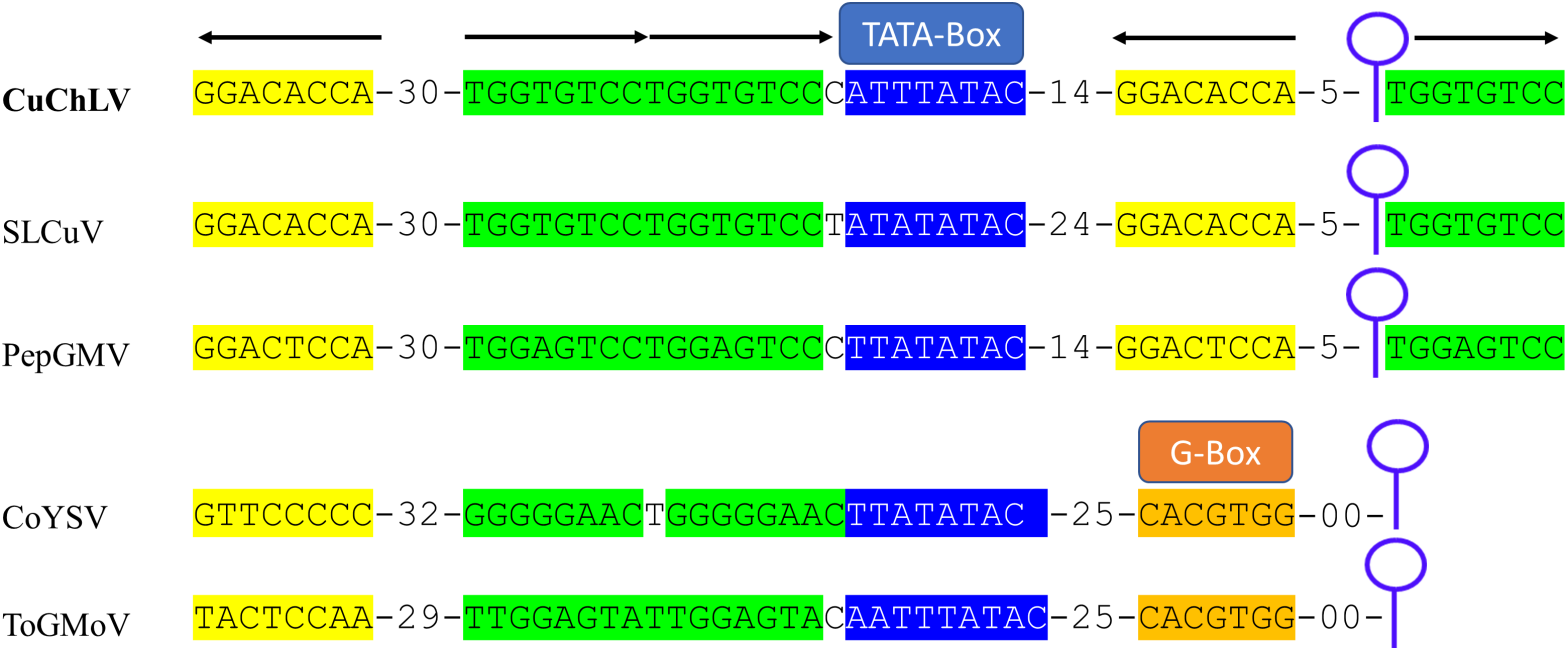
Analysis of replication origin structure of Cucumber chlorotic leaf virus. Comparison of the structure of the replication origin of CuChLV (MN013786) with the structure of begomoviruses of the SLCuV clade (SLCuV: M38183; PepGMV: MN013410) and two non-SLCuV BGVs (CoYSV: DQ875868; ToGMoV: MT083928). Iterons in a virion-sense (TGGTGTCC) are highlighted in green whereas iterons in a complementary-sense (GGACACCA) are highlighted in yellow. G-Box from typical BGVs is highlighted in orange. TATA-box is highlighted in blue. Arrows indicated the iterons sense. The stem-loop structure is shown as a blue line art.

The AC4 protein is 130 aa in length, which is unusually long since the average size of AC4 protein is ~85 aa for typical BGVs and 121 aa in length for BGVs belonging to SLCuV clade (Torres-Herrera et al., 2019). A BLASTP search revealed that only CuChLV and another three BGVs of the SLCuV clade have AC4 proteins with 130 aa (BLCrV: YP_009666510.1; *Wissadula yellow mosaic virus*: AOT83446.1; *Abutilon golden mosaic Yucatan virus*: YP_009506373.1). These findings suggest that CuChLV is a member of SLCuV clade. To corroborate this conclusion, we analyzed the replication origin, which is a distinctive feature of members of SLCuV clade (Argüello-Astorga et al., 1994). The analysis showed that the replication origin of CuChLV is similar to those belonging to SLCuV Clade (Fig. 3). In summary, CuChLV is a bipartite New World begomovirus with typical features of BGVs belonging to the SLCuV clade.

### Evolutionary history of CuChLV

**Figure 4:**
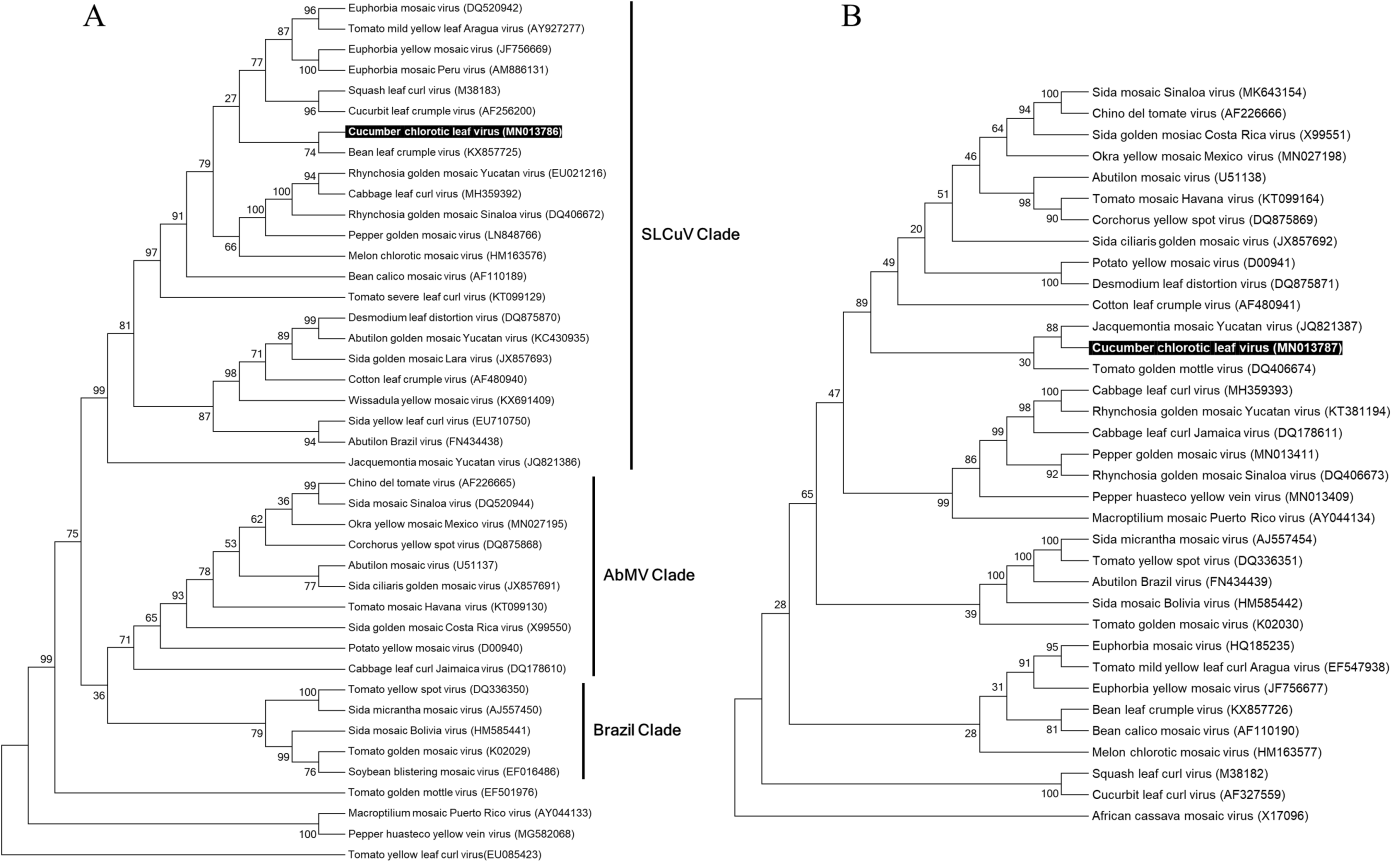
Phylogenetic relationships of Cucumber chlorotic leaf curl virus. Evolutionary histories of DNA-A (A) and DNA-B (B) of CuChLV were inferred by using the Maximum Likelihood method based on the General Time Reversible model. CuChLV name is highlighted in bold. Bootstrap values (1,000 iterations) are indicated for each node. GenBank accession numbers are indicated in brackets. Begomovirus clades are delimited by vertical lines. *Tomato yellow leaf curl virus* was included as an outgroup for the DNA-A analysis. DNA-B from *Africa cassava mosaic virus* was included as an outgroup for the DNA-B analysis.

In order to reconstruct the evolutionary relationship of CuChLV and establish if this virus is a true member of SLCuV clade, we performed a phylogenetic analysis using the maximum-likelihood method included in MEGA v10.04 package. Phylogenetic analysis for DNA-A of CuChLV displayed that this virus is grouped into the SLCuV clade and is closely related to BLCrV virus (Fig. 4A). CuChLV also is grouped into a subgroup of SLCuV clade that harbors to *Melon chlorotic mosaic virus* (MeCMV), the only virus that had been reported infecting cucumber on America continent until now. On the other hand, DNA-B was not grouped into the SLCuV clade, suggesting a component exchange event (Fig. 4A). However, DNA-B of CuChLV was close related to one member of SLCuV clade (*Jacquemontia mosaic Yucatan virus*). These findings confirm that CuChLV is a member of SLCuV clade.

To determine if CuChLV has a recombinant origin, we performed a recombination analysis using RDP4. First, we identified potential parents for CuChLV utilizing SWeBLAST employing a windows size of 250 nts. The search identified six potential parents (AF439402.1, MK256739.1, NC_043524.1, MH359390.1, AF224760.2 and AF130415.2) which were aligned using ClustalW. The alignment was later used in the RDP4 program. The recombination analysis showed that CuChLV is not a recombinant virus.

## Discussion

Findings provided here represent the first report of begomoviral infection affecting cucumber plants in North America. Seven BGVs infecting cucumber have been identified (Table S4). Most of these infections (six) were reported in the Old World, specifically in South Asia Table S4. In the Americas, there is only one record of begomoviral infections affecting cucumber (Romay et al., 2014). The infection registered in Venezuela was caused by MeCMV, a BGV of the SLCuV clade (Romay et al., 2014). Here we report that a cucumber field in Colima, Mexico, was affected by five species of BGVs from the New World (Fig. S3). The five species reported here are different from the seven previously reported (Table S4).

PhYVV and PepGMV are endemic and widely distributed species in Mexico (Pacheco et al., 1996). Both viruses have a wide range of reported hosts, about 8 and 16 species of plants, respectively, which does not include cucumber. These two viruses are often found co-infecting plants (Rodelo-Urrego et al., 2013), forming a viral complex that induces severe symptoms due to synergism (Rentería-Canett et al., 2011). Here we identified five cucumber plants infected with the complex PepGMV-PhYVV (Fig. S3). Thus, to our knowledge, this is the first report of PhYVV and PepGMV infecting cucumber plants.

ToGMoV is a BGV that is restricted to plants of the family *Solanaceae* such as *Solanum lycopersicum* and *Solanum rostratum* (Mauricio-Castillo et al., 2007) whereas RhGMSV is restricted to plants of the family *Fabaceae* such as *Glycine max* (Mauricio-Castillo et al., 2014) and *Rhynchosia minima* (Rodríguez-Negrete et al., 2019). The results obtained here show that these two BGVs are also capable of infecting plants of family *Cucurbitaceae*.

One of the five species of BGVs reported here is a novel species of BGVs. According to the ICTV species demarcation criteria (Brown et al., 2015), CuChLV is a new BGVs since the highest pairwise identity shared with other BGVs was 82.5%, which is below the threshold value of 91%. Phylogenetic analysis and genomic characterization showed that the novel virus is a New World BGVs belonging to SLCuV clade. This new species is the first BGV originally isolated from *C. sativus*. The seven previous BGVs species were isolated from other crops such as tomato (Mizutani et al., 2011; Shafiq et al., 2019), mungbean (Shahid et al., 2018), melon (Romay, Lecoq & Desbiez, 2015) and squash (Table S4).

Mixed infections with three or more begomovirus species were found in the six tested plants (Fig. S3), this suggests that cucumber is very susceptible to mixed infection of BGVs. This kind of infection in plants increases the likelihood of recombination, which is very common between BGVs. This molecular mechanism is an important driving force in evolution that facilitates the emergence of new virus strains and species of begomovirus (Padidam, Sawyer & Fauquet, 1999; Rojas et al., 2005). Although CuChLV was infecting all tested plants in combination with other BGVs, recombination analysis showed that CuChLV is not a recombinant virus. However, cucumber plants provide a high-risk environment for the emergence of new BGVs by recombination in the future. Thus, epidemiological consequences of the results reported here should be addressed.

At the time of writing this article, the genome sequence of a BGV isolated from an unidentified weed in Chiapas, Mexico was released (MN203175) (Alcalá-Briseño et al., 2020). When we compared the DNA-A of this virus with those of CuChLV, we found that both DNA-A share a high pairwise identity. Demarcation analysis showed that these two viruses belong to the same species since they share 97% nucleotide sequence identity. Since the CuChLV genome sequence was submitted and released first in the GenBank database, we suggest that the virus isolated in Chiapas is an isolate of CuChLV, according to the criteria established by the ICTV for species demarcation. The fact that CuChLV had been isolated in Colima and Chiapas suggests that this BGV is distributed throughout the region of Mexico known as the South Pacific which includes the states of Chiapas, Oaxaca, Guerrero, Michoacan, Colima and Jalisco.

Colima is a small state of Mexico, in which 28% of its territory is used for cropping (Instituto Nacional de Estadística y Geografía, 2016). This state is an important producer and exporter of, for example, cherry tomato, coconut, papaya, banana, cucurbits and lemon (Agricultural & Fisheries Information Service, 2018). Little information about phytopathogen effect on crops in Colima is available. To the best of our knowledge, previous to this work, five begomovirus (PhYVV; RhGMSV; *Tomato yellow leaf curl virus*; *Sida mosaic Sinaloa virus*; *Okra yellow mosaic Mexico virus*) have been reported in Colima, Mexico (Morales et al., 2005; Rodríguez-Negrete et al., 2019). Results presented here shown that Colima is rich in BGVs species. By adding the BGVs identified in this study with the previously reported viruses, now we can say that Colima has a diversity of eight species of BGVs, which represent 22% of the begomovirus reported in Mexico to this day.

## Conclusions

The begomoviral infection in a cucumber field in Colima, Mexico, was confirmed. Five species of begomovirus infecting the cucumber field were identified, one of them, CuChLV, is a new species that belongs to the SLCuV Clade. This new virus is the first begomovirus originally isolated from the cucumber. The viral infection documented here represents the first report of a begomoviral infection affecting cucumber crop in North America. Knowing the diversity of BGVs that affect cucumber crops in the Americas, will allow to establish strategies that permit counteract the effect of these phytopathogens in cucumber.

## Supplemental Information

10.7717/peerj.9245/supp-1 Supplemental Information 1 Primer mapping on partial genome of the potential new virus. Partial genome of probable new virus is composed by the partial DNA-A (A) and the partial DNA-B (B). The upper region of DNA-A (orange feature) was amplified with the specific primer F-Rep_PNA and R-CP_PNA. The lower region of DNA-A was amplified with the specific primers R-Rep_PNA and F-CP_PNA. These last combination also was used to detect the probable new virus in the six samples analyzed. The upper region of DNA-B (blue feature) was amplified with the specific primer F-BC1_NMB and R-BV1_NMB. The lower region of DNA-B was amplified with the specific primers R-BC1_NMB and F-BC1_NMB. Brown lines indicated the missing region.

10.7717/peerj.9245/supp-2 Supplemental Information 2 BLASTn hits distribution. Contigs from cucumber sequencing were compared against the nt database.

10.7717/peerj.9245/supp-3 Supplemental Information 3 Detection by PCR of BGVs identified by Illumina sequencing in the six cucumber samples. Detection by PCR of BGVs identified by Illumina sequencing in the six cucumber samples Lane “M” correspond to 1Kb DNA ladder (GeneRuler Thermo Scientific) and lane “N” correspond to negative reaction. A) Detection of PhYVV (F-ATAAAAACGCCATTCGCTGC/R-CCCGAAACAATGACACAATGG; 616 bp). B) Detection of PepGMV (F-AAGCTGTCATCGAAGTCGTC/R-CAACGTTCAAGCAGCCAAAG; 1,087 bp) C) Detection of RhGMSV (F-AACGGAACTCTCTGCTTGAC/RTCCTCCAGCATATAGCACTC;1,247 bp). D) Detection of ToGMoV (F-AGCTCCCTGAATGTTCGGATG/R-CCTGACCAACCAGAACATGAC;1,020 bp). E) PCR for CuChLV (F-TCTTGGTCAGAGACAGGAGAC/R-TCCTCCGTTTCAACTCTCCAC; 1,345 bp).

10.7717/peerj.9245/supp-4 Supplemental Information 4 Isolation of the complete genome of the potential new virus by PCR. The lane “M” corresponds to 1 Kb DNA ladder (GeneRuler Thermo Scientific). The samples loaded were from PCR reactions with primers F-Rep_PNA and R-CP_PNA (Lane 1), R-Rep_PNA and F-CP_PNA (Lane 2), R-BC1_NMB and F-BV1_NMB (Lane 3) and F-BC1_NMB and R-BV1 (Lane 4). The negative symbol indicates negative PCR reactions.

10.7717/peerj.9245/supp-5 Supplemental Information 5 Original gel used in the Fig. S4. The lane “M” corresponds to 1 Kb DNA ladder (GeneRuler Thermo Scientific). Lane 2 corresponds to the upper region of DNA-A (F-Rep_PNA/R-CP_PNA; 1507 bp). Lane 2 corresponds to the lower region of DNA-A (R-Rep_PNA/F-CP_PNA; 1345 bp). Lane 3 corresponds to the lower region of DNA-B (R-BC1_NMB/F-BV1_NMB; 1286 bp). Lane 4 corresponds to the upper region of DNA-B (F-BC1_NMB/R-BV1_NMB; 1448 bp)

10.7717/peerj.9245/supp-6 Supplemental Information 6 Original gels from the Fig. S3. Lane “M” correspond to 1Kb DNA ladder (GeneRuler Thermo Scientific) and lane “N” correspond to negative reaction. A) Detection of PhYVV (F-ATAAAAACGCCATTCGCTGC/R-CCCGAAACAATGACACAATGG; 616 bp). B) Detection of PepGMV (F-AAGCTGTCATCGAAGTCGTC/R-CAACGTTCAAGCAGCCAAAG; 1,087 bp) C) Detection of RhGMSV (F-AACGGAACTCTCTGCTTGAC/RTCCTCCAGCATATAGCACTC;1,247 bp). D) Detection of ToGMoV (F-AGCTCCCTGAATGTTCGGATG/R-CCTGACCAACCAGAACATGAC;1,020 bp). E) PCR for CuChLV (F-TCTTGGTCAGAGACAGGAGAC/R-TCCTCCGTTTCAACTCTCCAC; 1,345 bp).

10.7717/peerj.9245/supp-7 Supplemental Information 7 Assembled contigs.

10.7717/peerj.9245/supp-8 Supplemental Information 8 Begomoviruses contigs.

10.7717/peerj.9245/supp-9 Supplemental Information 9 List of primers used in this study.

10.7717/peerj.9245/supp-10 Supplemental Information 10 BLASTn results.

10.7717/peerj.9245/supp-11 Supplemental Information 11 Matrix score from demarcation analysis.

10.7717/peerj.9245/supp-12 Supplemental Information 12 Begomoviruses that affect cucumber crop.

## Additional Information and Declarations

### Field Study Permissions

The following information was supplied relating to field study approvals (i.e., approving body and any reference numbers): Alejandro Rodriguez, the person who gave verbal permission to access their land, is an independent farmer who is not affiliated with any company.

### DNA Deposition

The following information was supplied regarding the deposition of DNA sequences: The raw data of Illumina sequencing are available in Sequence Read Archive (SRX5908012). The complete genome sequence of the new virus is available at GenBank; DNA-A (MN013786.1) and DNA-B (MN013787.1).

### Data Availability

The following information was supplied regarding data availability: The raw data from Illumina sequencing are available at NCBI: SRX5908012. Assembled contigs are available in Data S1. Curated contigs are available in Data S2. BLASTn results are available in Table S2. The original gels for Fig. 3 are available in Fig. S3. Demarcation analysis results are available in Table S3. The original gels for Fig. S2 are available in Fig. S4. The complete genomes from CuChLV are available at NCBI: DNA-A (MN013786.1) and DNA-B (MN013787.1).

### New Species Registration

The following information was supplied regarding the registration of a newly described species: This proposed new species Cucumber chlorotic leaf virus has been submitted to the ICTV for consideration. This name is only valid and official after the ICTV has approved the name, and it has been ratified by the membership. [URL Pending].
