# Supplementary figures and images for: First report of begomoviruses infecting Cucumis sativus L. in North America and identification of a proposed new begomovirus species
Source: PeerJ. 2020 Jul 10;8:e9245. doi: 10.7717/peerj.9245 (PMC7357562; doi:10.7717/peerj.9245)

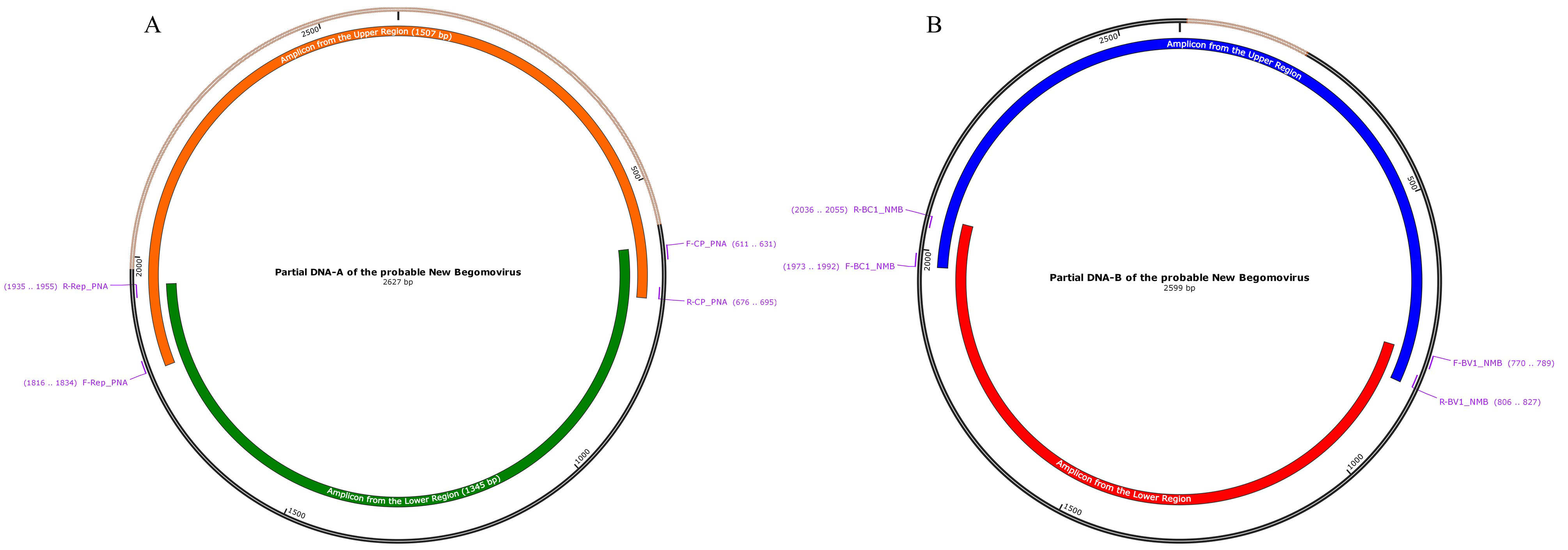

Supplement: Supplemental Information 1 — Partial genome of probable new virus is composed by the partial DNA-A (A) and the partial DNA-B (B). The upper region of DNA-A (orange feature) was amplified with the specific primer F-Rep_PNA and R-CP_PNA. The lower region of DNA-A was amplified with the specific primers R-Rep_PNA and F-CP_PNA. These last combination also was used to detect the probable new virus in the six samples analyzed. The upper region of DNA-B (blue feature) was amplified with the specific primer F-BC1_NMB and R-BV1_NMB. The lower region of DNA-B was amplified with the specific primers R-BC1_NMB and F-BC1_NMB. Brown lines indicated the missing region. [file peerj-08-9245-s001.png]

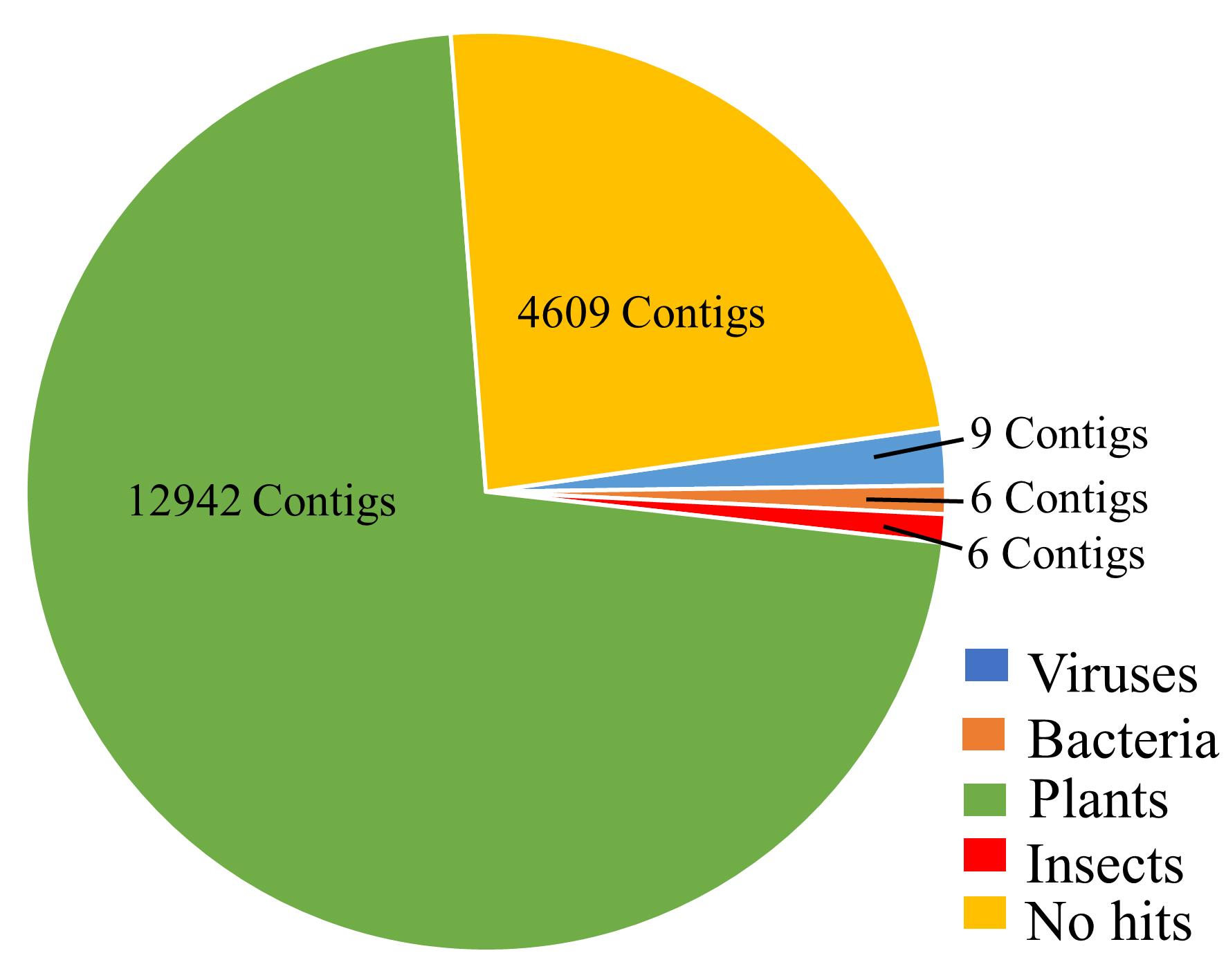

Supplement: Supplemental Information 2 — Contigs from cucumber sequencing were compared against the nt database. [file peerj-08-9245-s002.png]

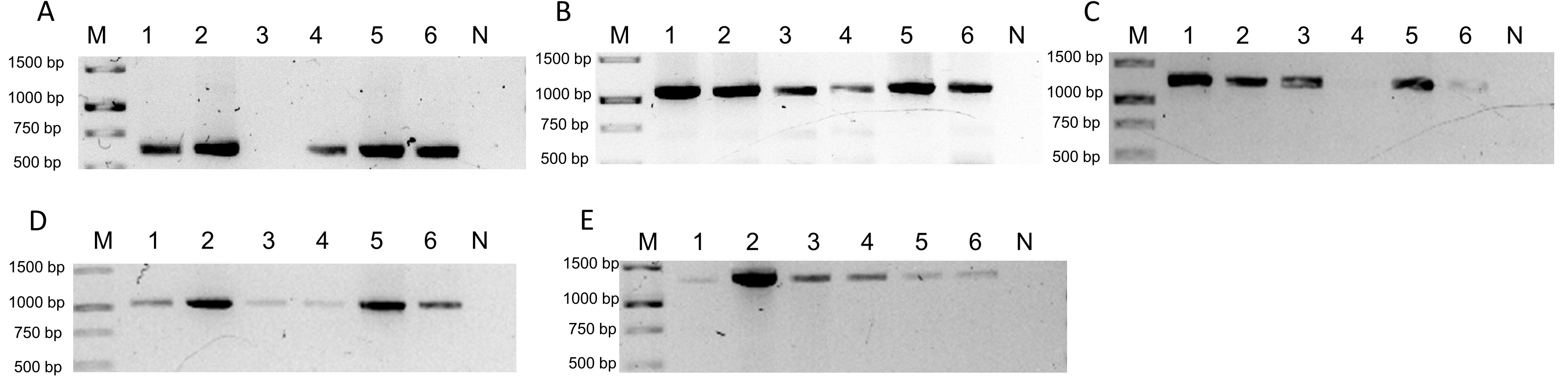

Supplement: Supplemental Information 3 — Detection by PCR of BGVs identified by Illumina sequencing in the six cucumber samples Lane “M” correspond to 1Kb DNA ladder (GeneRuler Thermo Scientific) and lane “N” correspond to negative reaction. A) Detection of PhYVV (F-ATAAAAACGCCATTCGCTGC/R-CCCGAAACAATGACACAATGG; 616 bp). B) Detection of PepGMV (F-AAGCTGTCATCGAAGTCGTC/R-CAACGTTCAAGCAGCCAAAG; 1,087 bp) C) Detection of RhGMSV (F-AACGGAACTCTCTGCTTGAC/RTCCTCCAGCATATAGCACTC;1,247 bp). D) Detection of ToGMoV (F-AGCTCCCTGAATGTTCGGATG/R-CCTGACCAACCAGAACATGAC;1,020 bp). E) PCR for CuChLV (F-TCTTGGTCAGAGACAGGAGAC/R-TCCTCCGTTTCAACTCTCCAC; 1,345 bp). [file peerj-08-9245-s003.png]

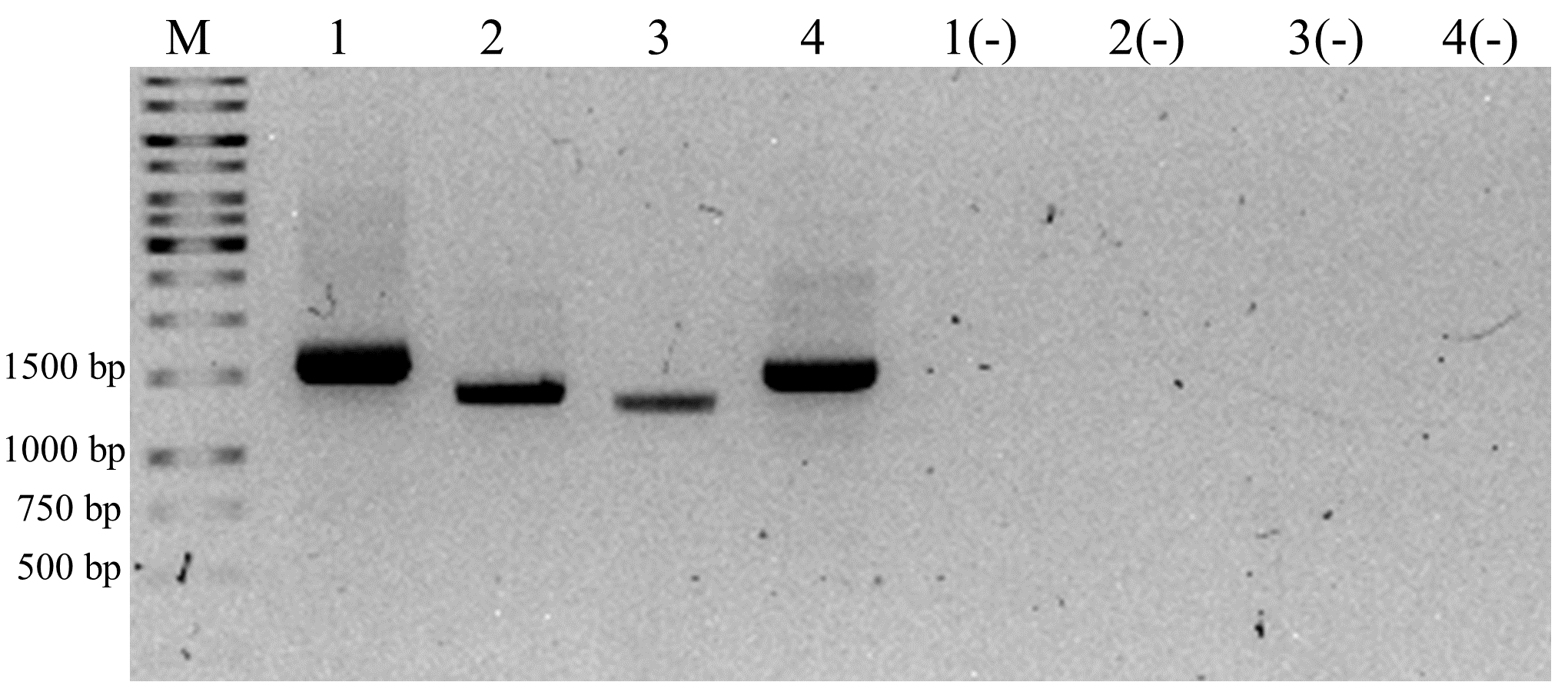

Supplement: Supplemental Information 4 — The lane “M” corresponds to 1 Kb DNA ladder (GeneRuler Thermo Scientific). The samples loaded were from PCR reactions with primers F-Rep_PNA and R-CP_PNA (Lane 1), R-Rep_PNA and F-CP_PNA (Lane 2), R-BC1_NMB and F-BV1_NMB (Lane 3) and F-BC1_NMB and R-BV1 (Lane 4). The negative symbol indicates negative PCR reactions. [file peerj-08-9245-s004.png]

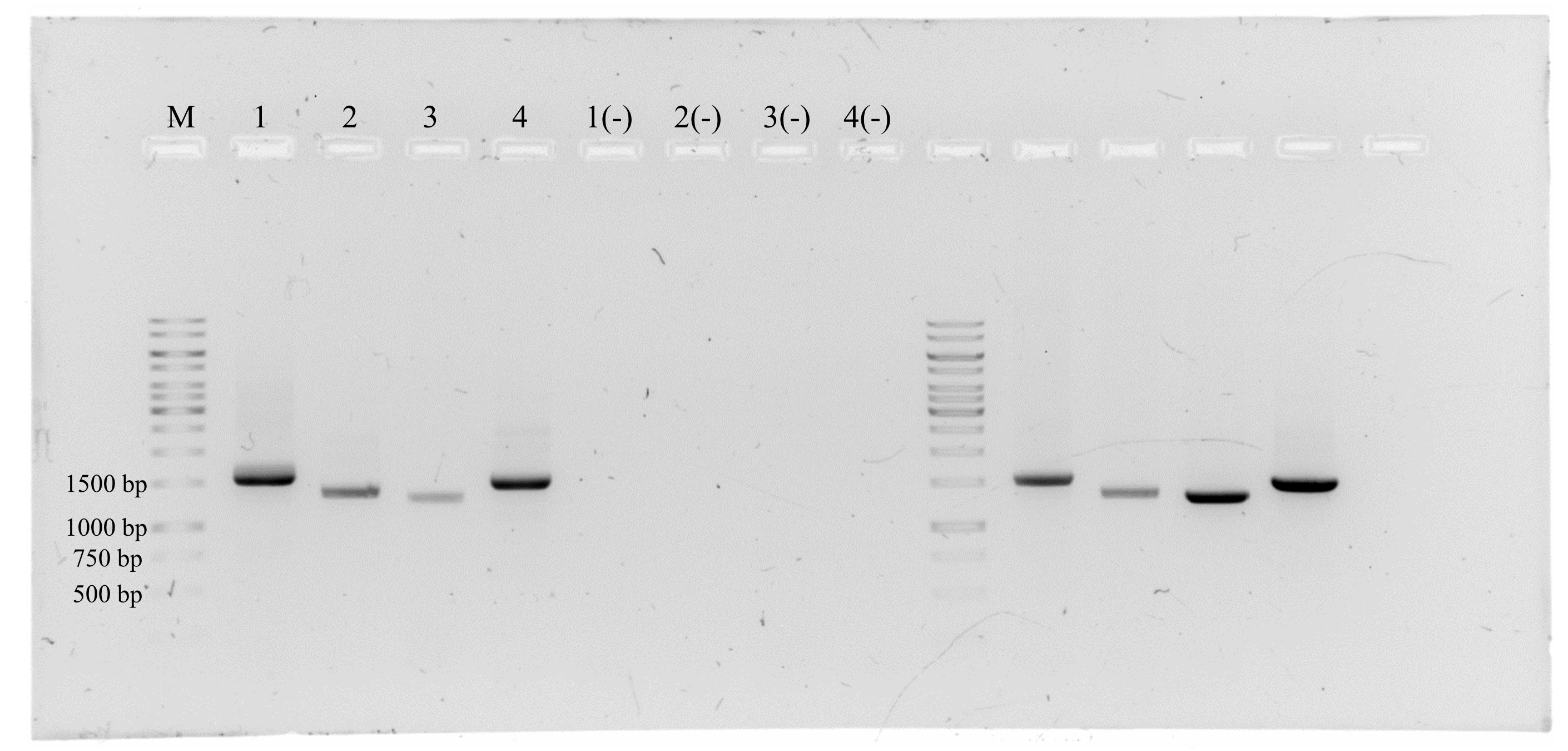

Supplement: Supplemental Information 5 — The lane “M” corresponds to 1 Kb DNA ladder (GeneRuler Thermo Scientific). Lane 2 corresponds to the upper region of DNA-A (F-Rep_PNA/R-CP_PNA; 1507 bp). Lane 2 corresponds to the lower region of DNA-A (R-Rep_PNA/F-CP_PNA; 1345 bp). Lane 3 corresponds to the lower region of DNA-B (R-BC1_NMB/F-BV1_NMB; 1286 bp). Lane 4 corresponds to the upper region of DNA-B (F-BC1_NMB/R-BV1_NMB; 1448 bp) [file peerj-08-9245-s005.png]

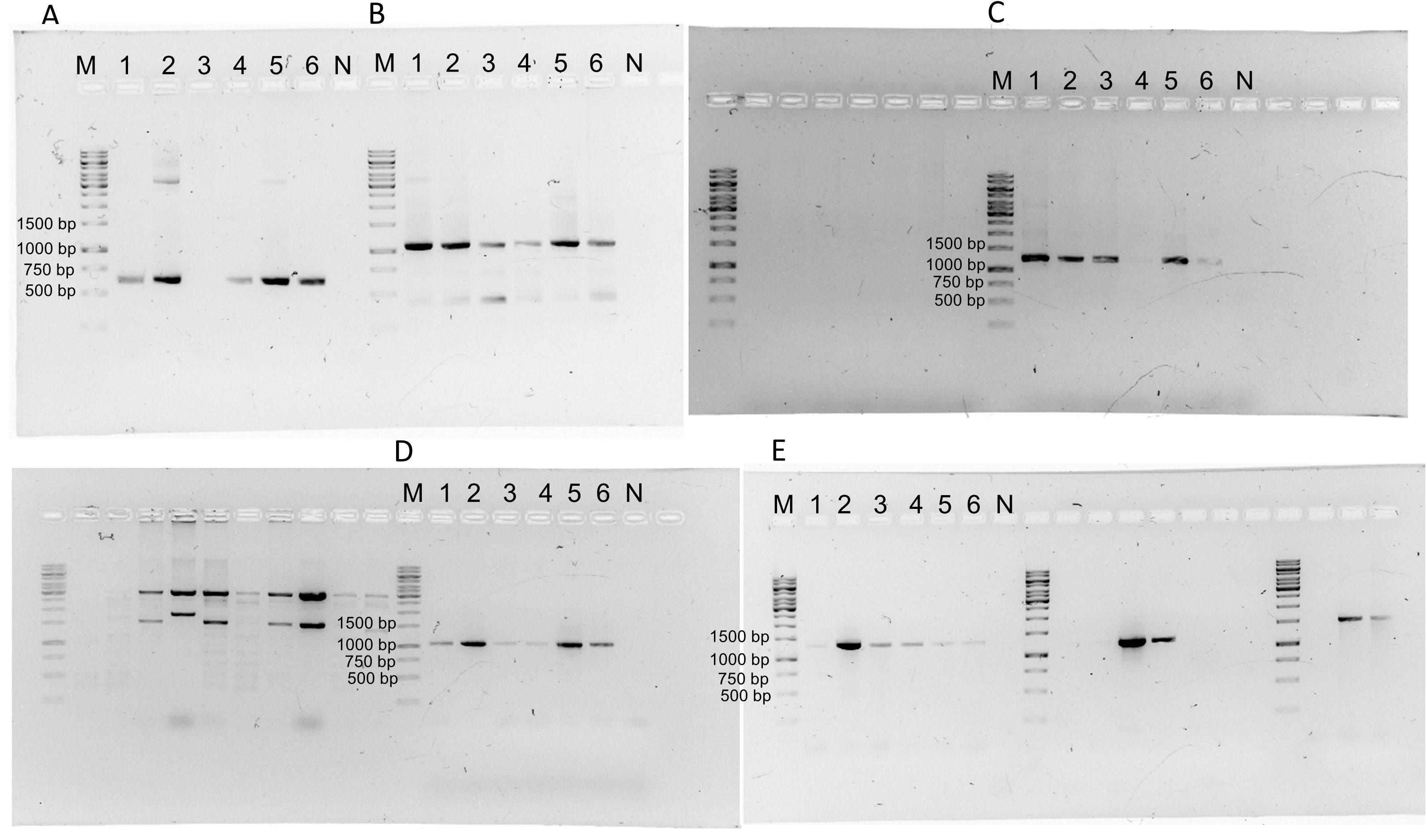

Supplement: Supplemental Information 6 — Lane “M” correspond to 1Kb DNA ladder (GeneRuler Thermo Scientific) and lane “N” correspond to negative reaction. A) Detection of PhYVV (F-ATAAAAACGCCATTCGCTGC/R-CCCGAAACAATGACACAATGG; 616 bp). B) Detection of PepGMV (F-AAGCTGTCATCGAAGTCGTC/R-CAACGTTCAAGCAGCCAAAG; 1,087 bp) C) Detection of RhGMSV (F-AACGGAACTCTCTGCTTGAC/RTCCTCCAGCATATAGCACTC;1,247 bp). D) Detection of ToGMoV (F-AGCTCCCTGAATGTTCGGATG/R-CCTGACCAACCAGAACATGAC;1,020 bp). E) PCR for CuChLV (F-TCTTGGTCAGAGACAGGAGAC/R-TCCTCCGTTTCAACTCTCCAC; 1,345 bp). [file peerj-08-9245-s006.png]
