# Supplementary material for: First report of begomoviruses infecting Cucumis sativus L. in North America and identification of a proposed new begomovirus species
Source: PeerJ. 2020 Jul 10;8:e9245. doi: 10.7717/peerj.9245 (PMC7357562; doi:10.7717/peerj.9245)
Supplement: Supplemental Information 9 [file peerj-08-9245-s009.docx]

**Table 1.** List of primers used in this study.

| Primer Name | Sequence | Target |
| --- | --- | --- |
| F-PhYVV | ATAAAAACGCCATTCGCTGC | Specific amplification of PhYVV |
| R-PhYVV | CCCGAAACAATGACACAATGG |  |
| F-PepGMV | AAGCTGTCATCGAAGTCGTC | Specific amplification of PepGMV |
| R-PepGMV | CAACGTTCAAGCAGCCAAAG |  |
| F-ToGMoV | ACCCGACCCGTTAGGTTAAA | Specific amplification of ToGMoV |
| R-ToGMoV | AACAGGGCATTCTCTGTGTG |  |
| F-RhGMSV | AACGGAACTCTCTGCTTGAC | Specific amplification of RhGMSV |
| R-RhGMSV | TCCTCCAGCATATAGCACTC |  |
| F-Rep_PNA | TTCCCGTCCTTGAATCACC | Specific amplification of the upper genome region of DNA-A CuChLV (region between nucleotide 2028-679) |
| R-CP_PNA | GCAGTGCTAGGTTCATTGTC |  |
| F-CP_PNA | TCTTGGTCAGAGACAGGAGAC | Specific amplification of the lower genome region of DNA-A CuChLV (region between nucleotide 614-1938) |
| R-Rep_PNA | TCCTCCGTTTCAACTCTCCAC |  |
| F-BV1_NMB | CTCTCGTGGTTGTGGTTGAC | Specific amplification of the lower genome region of DNA-B CuChLV (region between nucleotide 764-2049) |
| R-BC1_ NMB | CAGCTTTCTCATGACCTCAC |  |
| F-BC1_ NMB | CATGAACGACTCAGTCTTGC | Specific amplification of the upper genome region of DNA-B CuChLV (region between nucleotide 764-2049) |
| R-BV1_ NMB | GTATGTAGACATCCAGATGCAC |  |
