# Supplementary material for: First report of begomoviruses infecting Cucumis sativus L. in North America and identification of a proposed new begomovirus species
Source: PeerJ. 2020 Jul 10;8:e9245. doi: 10.7717/peerj.9245 (PMC7357562; doi:10.7717/peerj.9245)
Supplement: Supplemental Information 12 [file peerj-08-9245-s012.docx]

| **Begomovirus** | **Country** | **Region** | **GENEBANK ID** | **Reference** |
| --- | --- | --- | --- | --- |
| Cucumber chlorotic leaf virus | Mexico | N. America | MN013786 | This Work |
| Pepper huasteco yellow vein virus | Mexico | N. America | MN013408 | This Work |
| Pepper golden mosiac virus | Mexico | N. America | MN013410 | This Work |
| Tomato golden mottle virus | Mexico | N. America | MT083928 | This Work |
| Rhynchosia golden mosaic Sinaloa Virus | Mexico | N. America | MT083930 | This Work |
| Melon chlorotic mosaic virus | Venezuela | S. America | KF670626 | (Romay et al. 2014). |
| Mungbean yellow mosaic India virus | Oman | Asia | MF818045 | (Shahid et al. 2018) |
| Squash leaf curl virus | Lebanon | Asia | HM368373 | (Sobh et al. 2012) |
| Tomato leaf curl New Delhi virus | Indonesia | Asia | AB613825 | (Mizutani et al. 2011) |
| Tomato leaf curl Palampur virus | Iran: Jiroft, Kerman | Asia | JQ825226 | (Heydarnejad et al. 2009) |
| Tomato yellow leaf curl virus | Kuwaid | Asia | KR108214 | (Al-Ali et al. 2016) |
| Tomato leaf curl Palampur virus | Pakistan | Asia | MG252783 | (Shafiq et al. 2019) |

Table S4. Begomoviruses that affect cucumber crop.

References:

Mizutani T, Daryono BS, Ikegami M, Natsuaki KT. 2011. First Report of Tomato leaf curl New Delhi virus Infecting Cucumber in Central Java, Indonesia . Plant Disease 95:1485–1485.

Romay G, Lecoq H, Geraud-Pouey F, Chirinos DT, Desbiez C. 2014. Current status of cucurbit viruses in Venezuela and characterization of Venezuelan isolates of Zucchini yellow mosaic virus. Plant Pathology 63:78–87.

Shafiq M, Ahmad M, Nisar A, Manzoor MT, Abid A, Mushtaq S, Riaz A, Ilyas M, Sarwar W, Nawaz-ul-Rehman MS, et al. 2019. Molecular characterization and phylogenetic analysis of tomato leaf curl Palampur virus, a bipartite begomovirus, associated with Cucumis sativus L. in Pakistan. 3 Biotech. 9.

Shahid MS, Al-Mahmooli IH, Al-Sadi AM, Briddon RW. 2018. Identification of Mungbean yellow mosaic India virus infecting cucumber in Oman. Plant Disease. 102:465.

Heydarnejad J, Mozaffari A, Massumi H, Fazeli R, Gray A, Meredith S, Lakay F, Shepherd DN, Martin DP, Varsani A. 2009. Complete sequences of tomato leaf curl Palampur virus isolatesinfecting cucurbits in Iran. Archives of Virology. 154:1015–1018

Al-Ali E, Al-Hashash H, Ben Heji A, Al-Aqeel H. 2016. First Report of *Tomato yellow leaf curl virus* Infecting Cucumber in Kuwait. Plant Disease 100:656-656.

Sobh H, Samsatly J, Jawhari M, Najjar C, Haidar A, Abou-Jawdah Y. 2012. First Report of Squash leaf curl virus in Cucurbits in Lebanon. Plant Disease 96:1231-1231
